# Gene expression profiling and mechanism study of neural stem cells response to surface chemistry

**DOI:** 10.1093/rb/rbu012

**Published:** 2014-10-20

**Authors:** Ying Wang, Shenglian Yao, Qingyuan Meng, Xiaolong Yu, Xiumei Wang, Fuzhai Cui

**Affiliations:** ^1^Institute for regenerative medicine and biomimetic materials, School of materials science and engineering, Tsinghua University, Beijing 100084, China, ^2^Department of anatomy, histology and embryology, School of basic medical sciences, Capital Medical University, Beijing 100069, China and ^3^Department of material science and chemical engineering, Hainan University, Haikou 570228, China

**Keywords:** neural stem cell, chemical group, biomaterial, gene expression, receptor, signaling pathway

## Abstract

To declare the mechanisms of neural stem cells (NSCs) in response to material surface chemistry, NSCs were exposed to the self-assemble monolayers of alkanethiolates on gold surfaces terminated with amine (NH_2_), hydroxyl (OH) and methyl (CH_3_) for analysis. The morphological responses of NSCs were recorded; the gene expression profilings were detected by genechips; the gene expressions data of NSCs responded to different chemical groups were declared through the gene ontology term and pathway analyses. It showed that cells behaved dissimilar on the three chemical groups, the adhesion, proliferation and migration were easier on the NH_2_ and OH groups; the gene expressions of NSCs were induced differently, either, involved in several functional processes and signaling pathways. CH_3_ group induced genes enriched much in chemistry reactions and death processes, whereas many genes of cellular nucleotide metabolism were down-regulated. NH_2_ group induced NSCs to express many genes of receptors on membrane, and participated in cellular signal transduction of cell adhesion and interactions, or associated with axon growth. OH group was similar to NH_2_ group to induce the membrane response, but it also down regulated metabolism of cells. Therefore, it declared the chemical groups affected NSCs through inner way and the NH_2_, OH and CH_3_ groups triggered the cellular gene expression in different signaling pathways.

## Introduction

Neural stem cells (NSCs) have the capacities of self-renewal and differentiation into cell lines in neural system, such as neurons and glias, they are supposed to be the most potential means to substitute the lost cells and treat the injuries and degenerative diseases of nervous system [1, 2]. Therefore, controlling of NSCs fate for suitable utilization becomes a focus problem and attracts much more attentions.

Recent works show biomaterials or surrounding environments play key regulatory roles on NSCs fate determination by controlling their behaviors of adhesion, migration, proliferation and even differentiation [3–5]. Many factors of biomaterials are reported to involve in this process, such as stiffness, roughness, surface topography, chemistry, mechanics and micro- and nanopatterns [6–10]. In these intrinsic properties, the surface chemistry appears to a key role in cell–material interactions and cell regulation, which can dominate the cell biological process by modulating cellular responses, including survival, adhesion, migration, cell cycle progression and differentiation [10–13]. Many cells have been reported significantly influenced by it, including bone-derived cells [14], osteoblast [10], monocyte/macrophages [15] and so on. As regards NSCs, we also have shown some differences in their attachment, growth and even differentiation on chemical group’s surface [16]. As a result, much attention have been attracted recently to the potential use of surface chemical modification for its influence on cellular behaviors, and it is becoming increasingly important for biomaterials or environments design.

Despite of these extensive researches, there is still a lack of understanding of the mechanism of the impaction; it becomes highly desired to know how the chemical surface of materials regulates cellular behavior, especially for the stem cells regulation. It was more significant to NSCs for their great promise in neural repair. Any effective neurogenesis by NSCs therapy would require the understanding of mechanisms governing them proliferation and efficient differentiation. Therefore, research efforts focused on identifying the interactions between the NSCs and chemical surface and if and how they trigger the inner cellular process. That will be very important for understanding the mechanism of chemical surface actions, especially on NSCs. It will help to modulate the functionality of tissue-engineered cells and enable the design of more promising biomaterials to direct the fate of NSCs for neural repair applications. It is reported previously that cellular reactions to its environment are controlled by the variety of signal transduction processes. Various environmental stimuli initiate signal transmission processes in cell and then regulate their biological process. The behaviors of NSCs, such as adhesion, proliferation and division, are also regulated by many different signal transductions [17–20]. Many genes in cells change with the stimulation and involve in cellular regulation process.

Therefore, this study was performed to investigate the interactions and mechanisms of NSCs to surface chemistry properties, especially to reveal the gene expression difference and signaling pathways control. For this purpose, alkanethiol self-assembled monolayers (SAMs) terminated with methyl (CH_3_), amine (NH_2_) and hydroxyl (OH) were prepared as model. The cellular behaviors and the gene profiles of NSCs to the chemical surfaces, especially the membrane interactions and signaling pathway induction, were detected and analyzed by genechip.

## Materials and Methods

### SAMs preparation and characterization

SAMs of alkanethiols on gold were used to as model surfaces with well-defined chemistries. 1-Mercaptododecane [HS-(CH_2_)11-NH_2_], 1-dodecanethiol [HS-(CH_2_)11-CH_3_] and 11-mercapto-1-undecanol [HS-(CH_2_)11-OH] were purchased from Aldrich Sigma. Ethanolic alkanethiol solutions of 1.0 mM were prepared. Gold-coated 90 mm culture dishes were prepared by deposition of 50 nm gold films on 10 nm Ti films via an electron beam evaporator. Then, SAMs were assembled by immersing gold-coated substrates in ethanolic alkanethiol solutions for 4 h away from light. The SAMs of their respective alkanethiols were hereafter referred as CH_3_, NH_2_ and OH. The surface property of each SAM was analyzed by measuring density or roughness through goniometry and atomic force microscopy (AFM) (MFP-3D-S, Asylum Research, USA).

### NSCs culture on SAMs

The rat primary NSCs from embryo (purchased from Cyagen Biosciences Inc., Guangzhou, China) were cultured in serum-free media of D/F12 with 20 ng/ml epidermal growth factor (EGF), basic fibroblast growth factor (bFGF) and 2% B27 supplements. Then after being dispersed into single cells with syringe, the NSCs were cultured on CH_3_, NH_2_ and OH SAMs with the initial density of 10^6^ per dish (Φ = 100 mm) for 7 days; the cells on gold-coated dishes without alkanethiol served as control. The cells on the same kind of SAMs were cultured three times for biological replicates. The morphology and behavior of NSCs on SAMs were observed during the culture, photos were recorded by microscope (Leica).

### RNA extraction and microarray hybridization

Total RNA was extracted from NSCs on different SAMs using TRIzol reagent (Invitrogen) according to the instruction of manufactures. The RNA quality was assessed by Agilent 2100 bioanalyzer and RNA LabChip kits (Agilent). The samples pools of three independent biological replicates were mixed for gene expression analysis.

Chip of one-color microarray-based gene expression profile analysis (Agilent) was used and all the procedures were following the protocols from it. The total RNA was purified (QIAGEN RNeasy® Mini Kit) and then 2 μg RNA was converted into cDNA with a T7 RNA promoter primer. The cRNA was amplified and labeled with Spike-In Kit and One-Color Spike-Mix, the labeled/amplified cDNA was purified. This was then fragmented and hybridized to the genechip of 44 K microassays and incubated at 60°C for 17 h. The genechips were scanned on the GenePix 4000B scanners.

### The microarray data analysis

To identify differentially expressed genes, pairwise comparison analyses were preformed with analysis system using functions in R-package in R-software (https://www.r-project.org) and NCBI Entrez gene database. The genes of NSCs on experimental NH_2_, OH and CH_3_ SAMs were compared with that on control golden surface. The gene expression differences were identified with a stringent cutoff, the genes of at least one probe signal in the treatment and the control chip for the same gene showed parent, and only those up- or down-regulated genes exceeding the threshold of 3-fold change were selected for the further analysis. Only well-characterized genes in DAVID (Kyoto Encyclopedia of Genes and Genomes (KEGG)), EntrezGene, GenbankAccession, GenomicCoordinates, RefSeqAccession, and TIGRID database were included [21, 22]. The selected genes were preformed to identify gene ontology (GO) terms and KEGG pathways.

#### GO analyses

To assess the function and biological processes of the differentially expressed genes, the GO studies were adopted, which stated biologically information, including cellular location, molecular and biological function. This information explained the differences of NSCs on chemical group SAMs with respect to the control group. For each SAMs, the up- and down-regulated probe set identifiers were used as input and the enrichment was analyzed separately. The significantly enriched terms (*P* < 0.05), which reflects the enrichment in frequency, were preformed using R-package Fisher’s exact test and the testing correction (*q*-value) was performed using R-package John Storey’s method based on GO databases (http://www.geneontology.org/).

#### KEGG pathway analyses

To analyze the specific information about the signaling pathways being affected in treated conditions, pathway enrichment analyses were used to find several relevant pathways in response to the stimulation using the same algorithms as GO. It also allowed the identification of gene networks and how genes were regulated. The biological pathways mediated by differentially expressed genes were identified using KEGG pathway database (http://www.genome.jp/kegg/) and Biocarta database (http://www.biocarta.com), enrichment test of *P* < 0.05 was considered significant and selected. The enriched pathways involved in cell and extra-cellular interaction, signal transduction process and cellular biology process were taken into account.

## Results

### The properties of the alkanethiols SAMs

**Figure 1.**
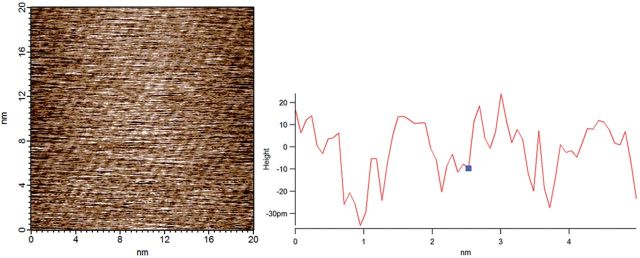
Image of surface morphology of region of SAMs by AFM. The alkanethiol SAMs of three chemical groups present the same regular structure feature with the homogeneous density.

The surface morphology of different alkanethiols SAMs on gold-coated substrate was performed by AFM. The images proved the regular alignments of gold atom with the same density, and the saturated alkanethiol monolayers for different groups connected on the gold atom nearly had same density as well, as shown in Fig. 1. It showed these SAMs assembled on gold were highly ordered and homogeneous, and provided well-controlled surface properties and the groups’ density. Other detail results such as contact angle and X-ray photoelectron spectrograph we reported in Deng *et al.* [23] and Zhi-Xu *et al.* [24].

### The morphology of NSCs on chemical group SAMs

**Figure 2.**
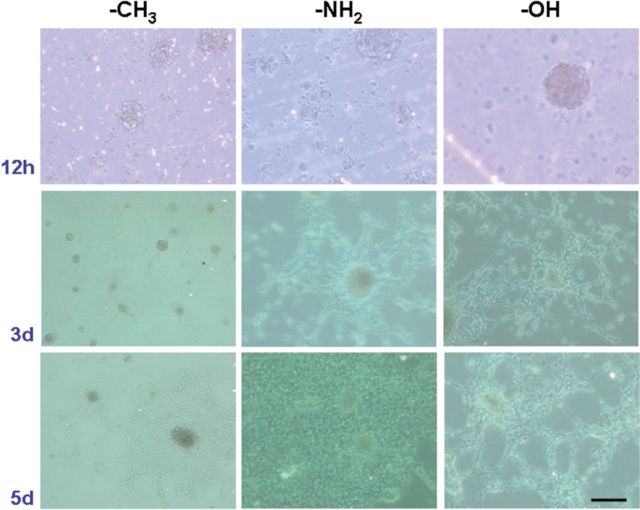
The adhesion and migration of NSCs on different chemical group SAMs. The NSCs cultured on all SAMs without adhesion at the first 12 h; to the 3rd day, most of NSCs adhered on NH_2_ and OH group SAMs, whereas on the CH_3_ SAMs, the cells which still clustered into many suspended neurospheres in the media. To the 5th day, on NH_2_ and OH SAMs, many cells migrated out form the adhesive clusters to a long distance and formed a network, especially on NH_2_ SAMs. Bar = 30 µm.

The cultured single NSCs on all group SAMs mostly clustered into small spheres without adhesion on the surface at the initial time; however, to the third day, NSCs adhered on NH_2_ and OH group SAMs significantly compared with that on the CH_3_ group SAM, which still clustered into many bigger neurospheres and suspended in the media. Furthermore, many cells on the NH_2_ and OH SAMs migrated out form the adhesive clusters to a long distance, and this migration was greatly enhanced with networks formation till the 5th day, especially on NH_2_ SAM. In contrast, the cells on CH_3_ SAM adhered gradually with little cells migration (Fig. 2). The NSCs on pure gold film control had no adhesion at all; the cells clustered into neurospheres and suspended in the media.

### The differentiation of NSCs on chemical group SAMs

Actually, in addition to the difference of adhesion and migration, the NSCs cultured on CH_3_, NH_2_ and OH SAMs were prone to differentiated into different phenotypes of neurons or glias by immunofluorescence and western blot. Briefly, it showed NSCs preferred to generate more neurons on CH_3_ group SAM; however, they differentiate into more glias on NH_2_ SAM. As to OH group, it dramatically promoted both neurons and glias differentiation, the detailed data were showed in our other submitted article to Nanoscale.

### The gene expression differences

#### Gene expression profiles

The NSCs cultured on the three SAMs showed different gene expression profiles by gene chip analysis, which may reflected the cellular mechanisms responded to the various chemical groups. To understand the mechanisms, the different genes met the selection conditions and expressed more than 3-fold changes were selected for further analysis. There were totally 150 up-regulated and 475 down-regulated genes for NSCs on CH_3_ SAMs; whereas that were 601 and 340 genes for NH_2_ SAMs and 154 and 45 genes for OH SAMs. These differently expressed genes were applied to further pathway and GO term analyses.

#### GO term enrichment analysis

To analyze the relation of different gene expression and cell behavior responded to the chemical groups, functional classification of differentially expressed genes was performed by GO term enrichment analysis. It helped to analysis the functions of genes. The up- and down-regulated genes of every chemical group were analyzed separately, and *P* < 0.05 was considered significant. For those much more genes enriched in a GO term, only the top 10 genes were listed in the table.

For cells on CH_3_ group SAM, many genes were up-regulated in the catalog ‘molecular functions’ associated with ‘drug and toxin binding’ function (Table 1). In term of ‘signal transducer activity’, several enriched genes were related to the chemicals binding; for example, ‘*TAAR1*’ was probably receptor for trace amines; ‘*Fabp1*’ contributed to bind long-chain fatty acids and other hydrophobic ligands; ‘*Olr*’ was a member of Olr receptors family for volatile amines. In the biological process, most associated genes contributed to the cellular response to the chemical stimulus. So it was apparent the genes of up-regulated were mostly associated with chemistry reactions and chemical signal transduction. Apart from this, some other genes also enriched in catalog ‘cellular component’, such as ‘*Myo1a*’ and ‘*Odf1*’, involving in cytoskeleton structures. In addition, some genes of cell killing also enriched, indicating the inactive impressions on cellular fate, which might support the phenomenon of their growth on the CH_3_ surface.

**Table 1.** GO term analysis of up-regulated genes on CH_3_ group SAM

GO terms	Hits	Gene symbol	Percent	*P* value
Molecular function				
Drug binding	3	*Alb*, *Fabp1*, *Hrh3*	4.05	0.0175
Toxin binding	1	*Alb*	33.33	0.0281
Signal transducer activity	33	*Wnt*, *Prlr*, *Olr*, *Corin*, *Taar*, *Amhr2*, *Vom2r*	1.15	0.0032
Cellular component				
Axoneme	2	*Pmfbp1*, *Odf1*	7.41	0.0179
Axoneme part	2	*Pmfbp1*, *Odf1*	20.00	0.0032
Apical part of cell	4	*Fabp1*, *Slc34a3*, *Myo1a*, *Acpp*	2.17	0.0452
Biological process				
Killing of cells of another organism	2	*Alb*, *Ifng*	28.57	0.0017
Behavior	7	*S100a8*, *Prok2*, *Alb*, *Cklf*, *Ifng*, *Hoxd10*, *Hrh3*	1.58	0.0421
Response to chemical stimulus	31	*Olr*, *Prf1*, *Hrh3*, *Aif1*	1.16	0.0039
Detection of stimulus	23	*Olr*, *Gngt1*	1.70	1.00E−04
Positive regulation of multi-organism process	1	*Ifng*	33.33	0.0281
Positive regulation of multicellular organismal process	5	*Aif1*, *Prok2*, *Alb*, *Ifng*, *Klks3*	2.13	0.0284

Notes: In GO 0042221, 31 genes were included, in which family Olrs were 28 totally, represented by ‘Olr’. Similar in GO 0051606, except ‘Gngt1’, all the other 22 genes were Olrs.

For down-regulated genes on CH_3_ group SAM, their GO terms enriched in functions related to nucleotide synthesis, organelle and cellular process of death and division (Table 2). Several hundreds of down-regulated genes enriched in term of ‘binding’ and ‘enzyme regulator activity’, regarding mainly the descent of nucleotide binding and synthesis functions. In addition, ‘chromosome segregation’ down-regulation also occurred in the biological process and some other important cellular process, including cell death, cell cycle and division, more than 100 genes participated in these functions, indicating the decrease in proliferation and division. Apart from this, the cellular component mainly about organelle was greatly down regulated, either. Moreover, the ‘cell projection’ term-related genes were down-regulated in abundant, reflecting the morphological results of seldom migration or movement for cell. The term of ‘caspase regulator activity’ included ‘*Bcl2a1d*’, ‘*Casp8ap2*’ was highly enriched either, which associated with cellular death.

**Table 2.** GO term analysis of down-regulated genes on CH_3_ group SAM

GO terms	Hits	Gene symbol	Percent	*P* value
Molecular function				
Nucleotide binding	96	*Atrx*, *Rock2*, *Eif2ak2*, *RGD1307234*, *Smc4*, *RGD1561537*, *Nlrc4*, *Hsp90*, *aa1*, *Matr3*, *Bub1*	5.09	0.0062
Nucleic acid binding	125	*Atrx*, *Zfp51*, *Zfp346*, *Znf606, LOC691257*, *LOC689296*, *Zfp52*, *Zfp40*, *RGD1565622*	5.41	2.00E−04
Chromatin binding	15	*Atrx*, *Zfp386*, *Mphosph8*, *Hells*, *Arid4a*, *Smarca1*, *Top2a*, *Smc1a*, *Pola1*	9.38	0.0027
Ribonucleoprotein binding	5	*Srp54a*, *Mtif2*, *Narg1*, *Srp72*, *Nol5*	15.15	0.0136
Ion binding	136	*Cyp11a1*, *RGD1305314*, *Mmp10*, *Car7*, *RGD1563278*, *Zfp52*, *Zfp51*, *Adam32*, *Rock2*, *Znf606*	4.83	0.0065
Caspase regulator activity	3	*Bcl2a1d*, *Casp8ap2*, *Xiap*	18.75	0.0332
Nucleoside-triphosphatase regulator activity	18	*Dock11*, *Sytl5*, *Myo9a*, *Chm*, *Rasa2*, *Iqub*, *Wdr67*, *Sytl5*, *Dock11*, *Tbc1d15*	6.67	0.0259
Cellular component				
Organelle lumen	70	*Prkdc*, *Zfp346*, *Esf1*, *Ccnb3*, *Smarca1*, *Matr3*, *Smc3*, *Iqub*, *Prpf39*, *Cops2*	5.15	0.015
Non-membrane-bounded organelle	105	*Stag2*, *Atrx*, *Pcm1*, *Rif1*, *Zfp346*, *Rock2*, *Ckap5*, *Esf1*, *Gria3*, *Smc4*, *Cenpe*	5.06	0.0049
Cell projection	39	*Pcm1*, *Gria3*, *Gria2*, *Ermn*, *Spp1*, *Itga1*, *Sema3a*, *Kif18a*, *Nov*, *Iqub*	5.64	0.0193
Biological process				
Multicellular organism reproduction	12	*Pla2g4a*, *Atp7a*, *Npy5r*, *Anxa1*, *Fgf7*, *Ptgs2*, *Zfx*, *Xdh*, *Kitlg*, *Angpt1*	7.32	0.0354
Microtubule-based process	19	*Kif5b*, *Rock2*, *Smc3*, *Ofd1*, *Hook3*, *Kif20b*, *Kif15*, *Cenpe*, *Kif18a*, *Pcm1*	8.26	0.003
Chromosome segregation	7	*Cenpf*, *Smc4*, *Top2a*, *Cenpe*, *Kif18a*, *Smc2*, *Brca1*	12.50	0.0094
Cell death	51	*Prkdc*, *Rb1cc1*, *Zfp346*, *Naip2*, *Eif2ak2*, *asp8ap2*, *Itga1*, *Alms1*, *Atp7a*, *Top2a*	5.47	0.0135
Cell cycle process	28	*Rock2*, *Smc4*, *Smc2*, *Smc3*, *Cenpe*, *Sycp2*, *Rad50*, *Pds5a*, *Taf1*, *Kif18a*	6.88	0.0045
Cell division	13	*Rock2*, *Aspm*, *Smc3*, *Pds5a*, *Top2a*, *Brca2*, *Cdc27*, *Ahctf1*, *Smc1a*, *Sycp2*	7.69	0.021

Accordingly, the presented GO terms indicated CH_3_ SAM had some interactions with the cellular surface by the way of amines or chemical signal detection and transduction, for several pathways about it were enriched. Additionally, the important biological process of cellular nucleotide metabolism was down-regulated and death process was up-induced.

For NSCs on NH_2_ group SAM, the pattern of GO term showed quite differently with that on CH_3_ group. Among the significant GO terms, several enriched terms associated with cells surface binding, extra-stimulation response and correspondingly the signal transduction in ‘biological process’, ‘molecular functions’ and ‘cellular component’ categories (Table 3 and Table 4). For those up-regulated genes (Table 3), the terms ‘cell surface’ and ‘amine binding’ were both enriched, including genes ‘*Fcer1a*’, ‘*Chrna7*’, ‘*Pecam1*’, ‘*Itgal*’, etc.; they involved in the interactions between NH_2_ group and receptors on cell surface, and participated in the amine binding process as well, which transmits chemical signals from outside the cell across the membrane to the inside of the cell. In addition, three genes ‘*Tat*’, ‘*Gucy1b2*’ and ‘*Gucy1a2*’ in terms of ‘cyclase’ and ‘lyase’ were participate in cellular signal transduction part of the G protein-signaling cascade. Other important biological processes were term ‘signal transducer activity’ mediated by 24 up-regulated genes, most of them were receptors on the cellular membrane. Furthermore, GO term ‘axon hillock’ was enriched; it seemed that NH_2_ triggered the growth of NSC axons. These functions were coincident to the cells adhesion and migration behavior. For those down-regulated genes, there were also enriched terms of cell surface and response (Table 4).

**Table 3.** GO term analysis of up-regulated genes on NH_2_ group SAM

GO term	Hits	Gene symbol	Percent	*P* value
Molecular function				
Cyclase activity	2	*Gucy1b2*, *Gucy1a2*	8.33	0.0096
Lyase activity	3	*Tat*, *Gucy1b2*, *Gucy1a2*	2.26	0.0433
Toxin binding	1	*Chrna7*	33.33	0.0227
Amine binding	3	*Htr1a*, *Tat*, *Chrna7*	3.13	0.0192
Channel regulator activity	2	*Kcns1*, *Chrna7*	4.44	0.0297
Signal transducer activity	24	*Ncr1*, *Chrna7*, *Olr376*, *Olr464*, *Olr783*, *Olr1243*, *Olr1264*, *P2ry13*, *Olr313*, *Vom2r56*	0.84	0.0348
Cellular component				
Cell surface	6	*Fcer1a*, *Chrna7*, *Pecam1*, *Itgal*, *Cd55*, *Tmprss11d*	1.78	0.014
Axon hillock	1	*Htr1a*	33.33	0.0227
Biological process				
Detection of stimulus	14	*Olr*	1.04	0.0232

Notes: In the analysis, the enriched GO terms of *P* < 0.05 were selected in the table, ‘hits’ was the number of hitted genes involved in the term, ‘percent’ was the ratio of hitted genes to the total genes in the GO term. The genes were list in the label by ‘genes symbol’, only the top 10 genes were listed when more than 10 genes were hitted according to the fold change.

**Table 4.** GO term analysis of down-regulated genes on NH_2_ group SAM

GO term	Hits	Gene symbol	Percent	*P* value
Cellular component				
Cell surface	5	*Il6*, *Art2b*, *Slc46a2*, *Kcnj3*, *Cd244*	1.48	0.0451
Biological process				
Multicellular organismal metabolic process	2	*Mmp10*, *Il6*	4.76	0.0258
Response to biotic stimulus	5	*Il6*, *Cyp2c7*, *Isg20*, *Nlrc4*, *Ccr1*	1.50	0.0437
Response to other organism	5	*Il6*, *Cyp2c7*, *Isg20*, *Nlrc4*, *Ccr1*	1.85	0.0201

**Table 5.** GO term analysis of up-regulated genes on OH group SAM

GO term	Hits	Gene symbol	Percent	*P* value
Molecular function				
Carbohydrate binding	17	*Colec10*, *Ctgf*, *Asgr1*, *Hbegf*, *Cyr61*, *Ccl3*, *Rpesp*, *Olr1630*, *Stbd1*, *Wbscr17*	5.20	3.00E−04
Peptide binding	8	*C5ar1*, *Calcr*, *Ccr5*, *Npy*, *Cckbr*, *F2rl2*, *Npy2r*, *Slc7a8*	3.92	0.0473
Cellular component				
Extracellular region part	33	*Wnt2*, *Vgf*, *Npy*, *Ctgf*, *Hbegf*, *Ccl*, *Cyr61*, *Cpz*, *Pyy*, *Nppb*, *Spn*, *Mfap5*	4.85	0
Extracellular space	23	*Vgf*, *Npy*, *Ccl*, *Hbegf*, *pyy*, *spn*, *Nppb*, *Il6ra*, *Edn1*, *Scg2*	4.72	1.00E−04
Extracellular matrix	11	*Wnt2*, *Spn*, *Cd44*, *Mmp28*, *Egfl6*, *Mfap5*, *Ctgf*, *Cyr61*, *Cpz*, *Col5a1*	4.53	0.0088
Cell surface	13	*C5ar1*, *Ccr5*, *Il6ra*, *Spn*, *Cd44*, *Il1rl1*, *Cav3*, *Tnfrsf12a*, *Itgb2*, *Cd83*	3.86	0.0154
Site of polarized growth	4	*Spn*, *Egfl6*, *Mfap5*, *Col5a1*	6.67	0.0322
Biological process				
Cell motion	27	*C5ar1*, *Ccr5*, *Hbegf*, *Ccl3*, *Fmnl1*, *Aif1*, *Itga11*, *Ptk2b*, *Ctgf*	4.58	0
Cell adhesion	23	*Spn*, *Dsg3*, *Myf5*, *Cobl*, *Itgb2*, *Egfl6*, *Cdh15*, *Ptk2b*, *Ctgf*, *Itga7*	4.14	6.00E−04
Cell death	27	*C5ar1*, *Nkx2-5*, *Prok2*, *Il6ra*, *Cckbr*, *Aldh1a3*, *Gch1*, *Aif1*, *Ptk2b*	2.90	0.0237
Cell proliferation	26	*Nkx2-5*, *Prok2*, *Edn1*, *Il6ra*, *Spn*, *Hbegf*, *Cckbr*, *Aif1*, *Itgb2*, *Ptk2b*	3.07	0.0138
Anatomical structure morphogenesis	44	*Prok2*, *Edn1*, *Scg2*, *Tmod1*, *Cobl*, *Itgb2*, *Thbs1*, *Ptk2b*, *Ctgf*, *Cyr61*	3.59	1.00E−04
Cell growth	11	*Wfdc1*, *Emp1*, *Cd44*, *Hbegf*, *Fbln5*, *Cav3*, *Ngf*, *Ptk2b*, *Ctgf*, *Cyr61*	6.29	8.00E−04
Positive regulation of growth	5	*Hbegf*, *Dio3*, *Ngf*, *Myod1*, *Ptk2b*	6.02	0.0251
Cell motility	25	*C5ar1*, *Ccr5*, *Hbegf*, *Ccl3*, *Aif1*, *Itgb2*, *Ptk2b*, *Ctgf*, *Ccl5*, *Cyr61*	5.03	0
Positive regulation of anti-apoptosis	3	*Il6ra*, *Cav1*, *Ptk2b*	10.71	0.02

**Table 6.** GO term analysis of down-regulated genes on OH group SAM

GO term	Hits	Gene symbol	Percent	*P* value
Molecular function				
Nucleotide binding	106	*Atrx*, *Prkg2*, *Smc4*, *Bub1*, *Kif20b*, *Dock11*, *Kif15*, *Cenpe*, *Kif18a*, *if2ak2*	5.62	0.0213
Nucleic acid binding	159	*Atrx*, *Zfp40*, *LOC689296*, *LOC691257*, *RGD1565622*, *Eif2ak2*, *Crop*, *Esco1*, *Zfp68*, *Ascc3*	6.88	0
Chromatin binding	17	*Atrx*, *Top2a*, *Zfp386*, *Hells*, *Arid4a*, *Pola1*, *Nsbp1*, *Smc1a*, *Mphosph8*	10.63	0.0021
Ribonucleoprotein binding	5	*Mtif2*, *Srp54a*, *Narg1*, *Srp72*, *Nol5*	15.15	0.0246
Cellular component				
Organelle lumen	88	*Mmrn1*, *Top2a*, *Kif20b*, *Iqub*, *Cenpf*, *Rad50*, *Kif11*, *Matr3*, *Esf1*, *Prkdc*	6.47	0.0012
Non-membrane-bounded organelle	135	*Atrx*, *Pcm1*, *Aspm*, *Smc4, Gria3*, *Gria2*, *Cenpe*, *Kif18a*, *RGD1308101*, *RGD1307234*	6.51	0
Organelle part	186	*Mmrn1, Atrx*, *Pcm1*, *Bub1*, *Kif15*, *Kif18a*, *Smc2*, *Stag2*, *Smc4*, *Aspm*	5.66	0.0016
Intracellular organelle part	184	*Mmrn1*, *Atrx*, *Cenpf*, *Aspm*, *Smc4*, *Bub1*, *Gria3*, *Gria2*, *Iqub*, *Kif20b*	5.64	0.0019
Postsynaptic density	8	*Exoc4*, *Akap5*, *Pja2*, *Grm3*, *Plcb4*, *Gria2*, *Gria3*, *Adam10*	11.94	0.017
Cell projection	43	*Pcm1*, *Kif18a*, *Gria3*, *Gria2*, *Iqub*, *Kcnma1*, *Ift74*, *Sema3a*, *Kif5b*, *Ccdc88a*	6.21	0.035
Asymmetric synapse	3	*Gria3*, *Gria2*, *Akap5*	25.00	0.026
Biological process				
Microtubule-based process	26	*Pcm1*, *Kif18a*, *Cenpe*, *Kif15*, *Kif20b*, *Kif11*, *Smc3*, *Rock2*, *Cenpj*, *Kif5b*	11.30	1.00E−04
Cell cycle	42	*Cenpf*, *Smc3*, *Smc4*, *Atm*, *Cenpe*, *Kif18a*, *Smc2*, *Rad50*, *Sycp2*	6.85	0.0095
Chromosome segregation	9	*Apc*, *Cenpf*, *Smc4*, *Spc25*, *Top2a*, *Cenpe*, *Kif18a*, *Smc2*, *Brca1*	16.07	0.0021
Cellular component organization	118	*Pcm1*, *Kcnma1*, *Smc4*, *Kif18a*, *Cenpe*, *Smc2*, *Top2a*, *Iqub*, *Eea1*, *Kif11*	5.64	0.0138
Cell cycle process	39	*Smc4*, *Cenpf*, *Kif11*, *Cenpe*, *Kif18a*, *Smc2*, *Rad50*, *Atm*, *Smc3*, *Sycp2*	9.58	0
Membrane docking	4	*Exoc4*, *Vcam1*, *Scfd1*, *Rock1*	16.67	0.0329
Cell division	15	*Aspm*, *Cep55*, *Smc3*, *Nuf2*, *Top2a*, *Brca2*, *Cp110*, *Smc1a*, *Sycp2*	8.88	0.0161
Establishment of organelle localization	7	*Copb1*, *Nlgn1*, *Exoc4*, *Myo5a*, *Cenpf*, *Kif18a*, *Syne1*	14.29	0.0111

It strongly suggested that NH_2_ group could induce active interactions with NSCs; it might bind to the receptors on cellular surface of NSCs and then triggered the signal transduction process, consequently promoted cells adhesion, migration and axons growth.

For NSCs on OH group SAM, a plenty of genes enriched in GO term ‘binding’ were down-regulated, including nucleic acid, chromatin and ribonucleoprotein binding (Table 6). Many genes involved in the DNA, RNA binding and related enzymes activities were down regulated, such as ‘*Atrx’*, ‘*Zfp*’, ‘*Smc4*’ for DNA binding; ‘*Srp54a*’ for RNA binding; ‘*Narg1*’ for ribosome and protein binding and polymerase ‘*Pola1*’, helicase ‘*Hells*’, protein kinase ‘*Prkg2*’ for enzyme activity. These genes associated with nearly the whole DNA duplication, RNA transcription and protein translation process. It appeared that the processes of DNA duplication and transcription, translation were down-regulated greatly to OH group surface. It might also be reasonable for the large portion of down-regulated genes in ‘cellular component’ enrichment. They made functions mostly in ‘organelle’, such as genes ‘*Mmrn1*’, ‘*Atrx*’ and ‘*Pcm1*’ were down for dozens of times, influencing the nucleotide binding and nuclease activity in regulation of transcription and translation. Additionally, in ‘biological process’ term, some genes such as ‘*Cenpe*’ and ‘*Aspm*’ played roles in negative regulation of neuron differentiation and neuroblast division was down expressed; some genes influenced the cellular structure and movement was down regulated either, such as ‘*Gria3*’ regulated dendritic shaft, ‘*Mmrn1*’ regulated microtubule motor activity and developmental process, ‘*Kif15*’ for microtubule-based movement.

Many genes of NSCs on OH group were up-regulated in the same time (Table 5). It could deduce these genes acted positively on the interactions of surface binding with the cell membrane, and correspondingly enhanced the adhesion, growth and motion.

Overall, the genes expression profiling showed the OH group could interact with cell membrane and promoted adhesion, growth and especially cell migration. Meantime, it influenced the cell circle by reducing the process of replication, transcription and translation.

#### KEGG pathway analysis

KEGG pathways analyses were used to assess the statistical significant pathways associated with differentially expressed genes. The up- and down-regulated genes of NSCs on the three chemical groups were analyzed, respectively. Probes were mapped to genes identifiers and gene identifiers were used as the input in the statistical analysis; *P* < 0.05 was considered significant. Only those pathways associated with cellular interaction, metabolism and biological process were included.

For CH_3_ group, the enrichment analysis revealed that 12 pathways were associated with up-regulated genes and 18 pathways were significant in down-regulated genes, as listed in Table 7. Analysis of functions showed pathways mediated cellular adhesion and growth processing were significantly down-regulated, such as ‘focal adhesion’, ‘axon guidance’ and ‘cell cycle’ pathways. However, some pathways mediated chemical signal detection and transduction, chemical drug metabolism and rejection reaction were found up-regulated. In addition, three signaling pathways associated cell growth and differentiation were triggered as well, such as JAK-STAT and TGF-β signaling pathway, indicating the regulation on the cells biological process.

**Table 7.** Pathway analysis of up- and down-regulated genes on CH_3_ group SAM

Regulation	Pathway	Hits	Percent	*P* value
Down	Non-homologous end-joining	4	30.77	2.00E−04
	NOD-like receptor signaling pathway	9	13.85	0
	Homologous recombination	3	11.54	0.0149
	Inositol phosphate metabolism	5	8.77	0.0051
	Cell cycle	11	8.33	1.00E−04
	Axon guidance	10	7.46	3.00E−04
	RNA degradation	4	6.56	0.0296
	Phosphatidylinositol signaling system	5	6.49	0.0162
	VEGF signaling pathway	5	6.25	0.0187
	Spliceosome	8	6.06	0.0039
	Gap junction	5	5.75	0.0253
	Oocyte meiosis	6	5.17	0.0232
	Apoptosis	5	5.05	0.0399
	CAMs	7	4.43	0.0301
	Neuroactive ligand–receptor interaction	13	4.00	0.0088
	Focal adhesion	8	3.94	0.0378
Up	Graft-versus-host disease	3	5.00	0.0012
	Allograft rejection	3	4.84	0.0013
	Hedgehog signaling pathway	2	3.85	0.014
	Drug metabolism—other enzymes	2	3.70	0.015
	Metabolism of xenobiotics by cytochrome P450	2	2.82	0.0247
	Phosphatidylinositol signaling system	2	2.60	0.0286
	TGF-beta signaling pathway	2	2.35	0.0341
	ErbB signaling pathway	2	2.22	0.0378
	Jak-STAT signaling pathway	3	2.01	0.0144

Notes: the enriched signal pathways were listed following the descending order of ‘percent’. The ‘percent’ was ratio of the hitted genes to the total genes in the pathway. The ‘hits’ meant the number of hitted genes in the pathway.

For NH_2_ group, the pathways on cellular adhesion and interactions with membrane receptors were up-regulated apparently, indicating the active binding and recognition to cells. Whereas the down-regulated pathways, such as ‘NOD-like receptor signaling pathway’ and ‘Graft-versus-host disease’, indicated the immune and rejection responses to NH_2_ were decreased (Table 8). Therefore, it could deduce NH_2_ group was easily accepted by NSCs.

**Table 8.** Pathway analysis of up- and down-regulated genes on NH_2_ group SAM

Regulation	Pathway	Hits	Percent	*P* value
Down	NOD-like receptor signaling pathway	3	4.62	8.00E−04
	Graft-versus-host disease	2	3.33	0.0119
	Drug metabolism–cytochrome P450	2	2.41	0.0215
	Calcium signaling pathway	4	2.09	0.0018
	Neuroactive ligand–receptor interaction	4	1.23	0.0115
	Cytokine–cytokine receptor interaction	3	1.22	0.0287
Up	Fc epsilon RI signaling pathway	3	3.70	0.0015
	Gap junction	2	2.30	0.0239
	CAMs	3	1.90	0.0094
	Neuroactive ligand–receptor interaction	5	1.54	0.002

For OH group (Table 9), several pathways mediated cellular adhesion, proliferation and differentiation processing were up-regulated significantly, including ‘ECM–receptor interaction’, ‘Focal adhesion’, ‘ErbB signaling pathway’, ‘TGF-beta signaling pathway’, ‘Hedgehog signaling pathway’, ‘Jak-STAT signaling pathway’ and so on. However, for down-regulated genes, some pathway associated metabolism processes were enriched, such as ‘Protein export’, ‘RNA degradation’ and ‘mTOR signaling pathway’. In addition, there were still some functions about the down-regulated pathways of ‘Axon guidance’, ‘CAMs’, ‘Neuroactive ligand–receptor interaction’; it suggested the complex networks in the same pathway on OH group, some genes were up-regulated, meanwhile others were down.

**Table 9.** Pathway analysis of up- and down- regulated genes on OH SAM

Regulation	Pathway	Hits	Percent	*P* value
Down	Non-homologous end-joining	3	23.08	0.0042
	NOD-like receptor signaling pathway	9	13.85	0
	Mismatch repair	3	13.64	0.015
	Homologous recombination	3	11.54	0.0225
	Cell cycle	13	9.85	0
	RNA degradation	6	9.84	0.0028
	Phosphatidylinositol signaling system	6	7.79	0.0081
	mTOR signaling pathway	4	7.02	0.0393
	Axon guidance	8	5.97	0.0105
	T cell receptor signaling pathway	6	5.22	0.0427
	CAMs	8	5.06	0.0245
	Neuroactive ligand–receptor interaction	13	4.00	0.0276
Up	ECM–receptor interaction	8	9.88	0
	Focal adhesion	14	6.90	0
	Hedgehog signaling pathway	3	5.77	0.0128
	Neuroactive ligand–receptor interaction	16	4.92	0
	TGF-beta signaling pathway	4	4.71	0.0081
	Cytokine–cytokine receptor interaction	11	4.47	0
	Chemokine signaling pathway	7	3.91	0.0014
	Axon guidance	5	3.73	0.008
	Tight junction	5	3.70	0.0083
	Drug metabolism–cytochrome P450	3	3.61	0.0409
	ErbB signaling pathway	3	3.33	0.0497
	CAMs	5	3.16	0.0152
	Regulation of actin cytoskeleton	7	3.14	0.0046
	Calcium signaling pathway	6	3.14	0.0084
	Jak-STAT signaling pathway	4	2.68	0.0474

**Figure 3.**
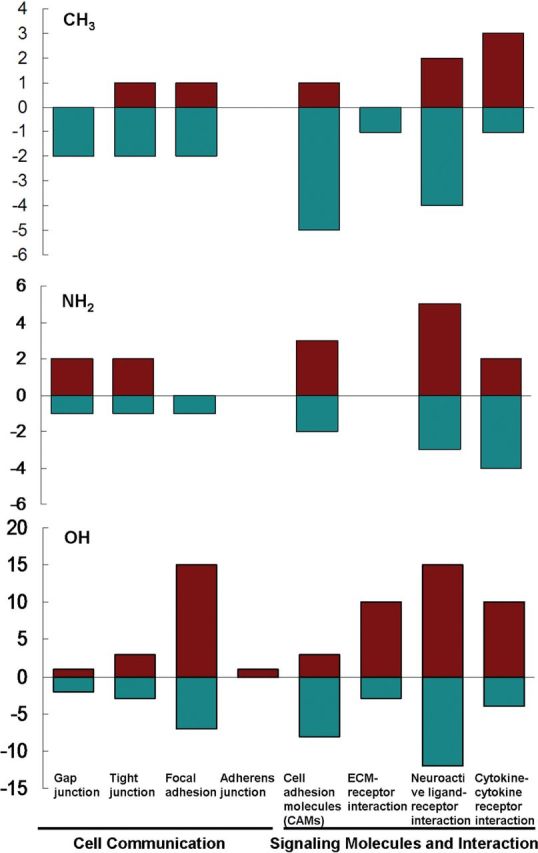
The number of differential gene in pathways for NSCs responded to chemical groups. The categories of cell communication and signaling molecules and interaction included eight pathways as shown in the figure presented by eight columns. The number of gene in each pathway presented in ‘*y*’ axis; the red columns meant the up-regulated genes; the green ones meant the down-regulation. It showed the tendency of interactions of chemical surface with the cell on CH_3_ group, the pathways were mainly down-regulated and implied the negative interactions to the cell. On the contrary, they were mostly up-regulated on NH_2_ and OH groups, indicating the extensive interactions with the cell.

**Figure 4.**
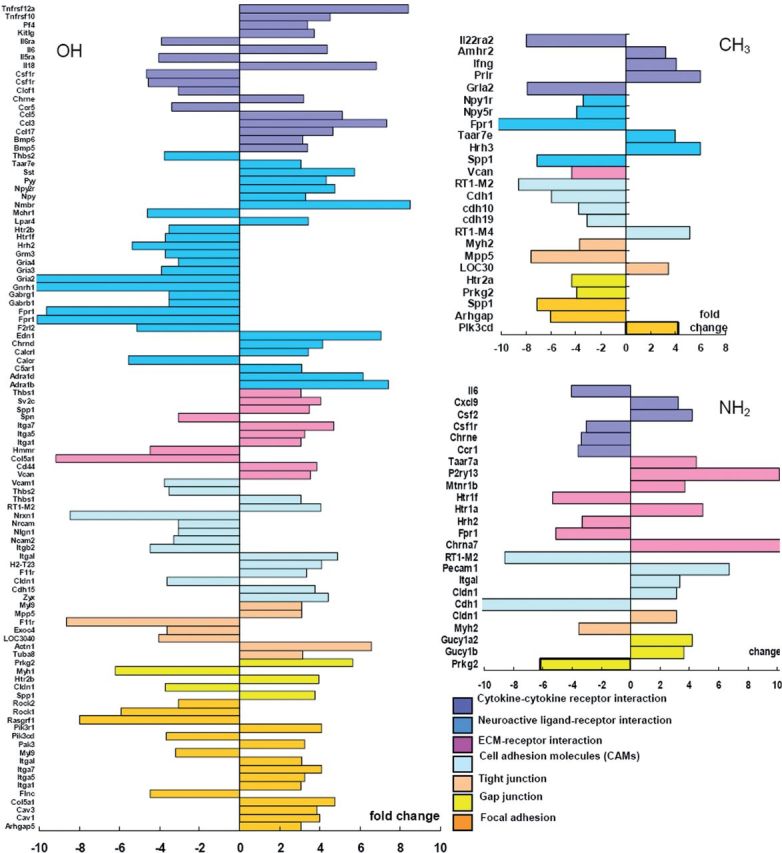
The differential gene expression in cell communication and signaling molecules and interaction pathways. The column showed the fold change of each gene in the two pathways. The genes with the same color were in a same catalog as shown.

The genes expression profiles showed the different ways that chemical groups acted on the cell. In all differential genes in pathways, those involved in cellular communication and signal interactions were analyzed extensively, as shown in Fig. 3 and Fig. 4. It showed the genes for adhesion and membrane receptors were usually down-regulated for CH_3_ group, for example, gene ‘*cdh*’, ‘*vcan*’ and ‘*spp*’, which mediated cell-to-cell and cell-to-matrix interactions with lowered expression. In contrast, for OH and NH_2_ group, many genes encoded membrane receptors and cell adhesive molecules were highly expressed, promoting cell adhesion and interactions to ECM or other cells. For example, on NH_2_ group, the NSCs expressed ‘*Itg*’, ‘*pecam*’ genes, and that was similar for OH group, the genes such as ‘*Itg*’, ‘*Cdh*’ and ‘*Thbs2*’, were up-regulated. These genes encoded the typical membrane receptors and mediated cell–matrix interaction. Furthermore, these two groups might also work on NSCs through neuroactive ligand–receptor interaction pathways, for many genes in this pathway expressed high.

In addition, all the three chemical groups could act on cell through cytokine–cytokine receptor interaction pathways, many genes in this way were up or down regulated and possibly involved in a wide variety of biological functions, especially in regulating survival, proliferation and differentiation of cells.

## Discussion

As most used and efficient method, surface chemistry dramatically developed the materials as well as the chemical engineering technology. Moreover, it was recently found had various functions on cellular biological process [10, 14, 25, 26], even on stem cell regulation [27, 28]. Although it opens up many possibilities for designing biomaterials, and even cellular engineering, it is still restricted by the limited understanding of biological reactions to surface chemistry. Therefore, a better understanding of these mechanisms will be crucial to tight regulation of these properties for controlling the cells, especially for NSCs fate to suitable regenerative efforts; and to the development of biomaterials for neural repair applications, which was becoming increasingly concerned along with the knowledge of too much difficulty to accomplish self-repair following neural system injuries [29].

Chemical groups were widely used as surface functionality. It was reported chemical groups made some functions on cell adhesion, stem cell maintain, materials endothelialization [27, 30–32]. Our results confirmed the functions of chemical groups on NSCs and demonstrated they could modulate the cellular inner gene expression in biological process. The results provided gene expression profiling and genes reacted to the chemical groups by ways of cellular signaling transduction, including binding, shape, cell cycle, growth and so on. Moreover, the different chemical groups might affect the cellular process through different pathway, including cell–matrix interactions and signal transductions pathways. These results would help to know the mechanisms of surface chemistries on NSCs regulation.

The results of morphology showed NH_2_ and OH group promoted NSCs adhesion, migration and growth; this phenomenon matched their gene expressions. It revealed that cells had active communications with the two groups with many genes encoded membrane receptors and molecules in signaling pathways highly expressed. NH_2_ and OH groups could tend to bind the cell’s surface for cell adhesion, some molecules, such as cadherins for mediating cell–cell adhesion, were highly up-expressed. Moreover, the results suggested NH_2_ and OH group probably acted on NSCs by way of ECM–receptor interactions through the membrane receptors such as integrins and pecam, and then triggered the signaling pathways to promote the cellular adhesion, migration and growth, for some pathways of cell adhesion, neuroactive ligand receptor interactions were up regulated. Especially, it seemed that the OH group had more complex interactions with the NSCs, for a part of the moleculars for adhesion and ligands might not involve in the interactions with the cells; and it not only interacted with cell surface but also could act as an extracellular component to involve in the extracellular environment and then have further impression on cellular process. As a result, many signaling pathways for stem cell proliferation and maintenance were also highly expressed, such as TGF-beta signaling, Jak-STAT signaling, ErbB signaling and Hedgehog signaling pathway [33–36]. The difference of the groups induced different cellular responses through several ways. The NH_2_ and OH groups had strong hydrophilicity, this property might made them had more interactions with cell’s surface. Meantime, some genes for amine and peptides binding were also up-expressed on NH_2_ and OH groups surfaces, the binding might adsorb and bind the special proteins in the media and then promote the cells adhesion and migration indirectly, as showed in the report of endothelial cell growth on chemical groups [37].

Conversely, the NSCs were not easy to adhere on CH_3_ group surface. Many cells still formed clusters till the 5th day of culture, only a little part of NSCs migrated out on the surface. GO term analysis showed CH_3_ group played a role like a normal chemical agent with the cells, without so many interactions with membrane receptors. Accordingly, the signaling pathways like focal adhesion, cell adhesion molecules (CADs), neuroactive ligand–receptor interaction and axon guidance were all down expressed. Although some signaling pathways were activated including TGF-beta signaling, Jak-STAT signaling, ErbB signaling, which especially being required for stem cell maintenance [33–35]. And thereby, the reproduction and division of cell were depressed as showed in gene expression. Therefore, CH_3_ group could help NSCs to maintain an undifferentiated state and keep the stem cell properties.

## Conclusions

The information presented in his study declared of interactions with the cellular receptors and cellular biology process regulation by chemistry property. It showed the different gene expressions associated with biological functions of membrane interactions, adhesion, proliferation and so on, through several different signaling transduction pathways. NH_2_ and OH groups had active interaction with cell through the cellular adhesion molecules and membrane receptors, then triggered the signaling pathways of adhesion, migration, proliferation and division; CH_3_ group had less interactions through the membrane receptors, it intended to maintain the property of NSCs. It was helpful to know the molecular mechanism of cellular chemistry controlling and should be useful for the development of biomaterials to regulate the preservation, proliferation and differentiation of NSCs.
